# Reviewed article: Tapajós AS, Oliveira LP, Amaral AHM, Catete CP, Santos WS, Garcez LM. Canine leishmaniasis in the urban area of a municipality in the Amazon, Brazil: a cross-sectional study on prevalence, distribution and phlebotomine fauna during the dry season, 2023. Epidemiol Serv Saude. 2025:34;20240130.a

**DOI:** 10.1590/S2237-96222025v34e20240130.a

**Published:** 2025-06-13

**Authors:** Kellyn Kessiene Cavalcante

**Affiliations:** Universidade Federal do Ceará, Saúde Comunitária

**Table:** 

Rating	Excellent	Good	Average	Below average	Poor
Interest			✓		
Quality				✓	
Originality			✓		
Overall			✓		

**Table:** 

Questionnaire	Yes	No	Not applicable
Does the manuscript contain new and significant information to justify publication?		✓	
Does the Abstract (Summary) clearly and accurately describe the content of the article?		✓	
Is the problem significant and concisely stated?		✓	
Are the methods described comprehensively?		✓	
Are the interpretations and conclusions justified by the results?	✓		
Is adequate reference made to other work in the field?	✓		

## Manuscript Structure

Length of article is: Too short

Number of tables is: Too few

Number of figures is: Too few

Please state any conflict(s) of interest that you have in relation to the review of this paper (state “none” if this is not applicable): None

## Comments

O manuscrito precisa de muitos ajustes para a publicação. Seguem algumas sugestões:

INTRODUÇÃO: Contextualizar de forma mais focada no tema. Sem necessidade do primeiro parágrafo.

OBJETIVO: Precisa ser revisado. Não está relacionado ao título.

MÉTODOS: Inserir versões dos softwares utilizados para a distribuição espacial; melhorar a descrição do passo-a-passo da pesquisa.

RESULTADOS: Figura 1 – Inserir rótulos dos eixos do gráfico.

DISCUSSÃO: Melhorar a escrita, trazendo abordagens de outros autores com foco nos seus achados; o primeiro parágrafo deve trazer os pontos mais relevantes.

